# Resveratrol: Multi-Targets Mechanism on Neurodegenerative Diseases Based on Network Pharmacology

**DOI:** 10.3389/fphar.2020.00694

**Published:** 2020-05-14

**Authors:** Wenjun Wang, Shengzheng Wang, Tianlong Liu, Yang Ma, Shaojie Huang, Lu Lei, Aidong Wen, Yi Ding

**Affiliations:** ^1^Department of Pharmacy, Xijing Hospital, Fourth Military Medical University, Xi'an, China; ^2^Department of Pharmacy, Shaanxi University of Chinese Medicine, Xi'an, China; ^3^Department of Medicinal Chemistry, School of Pharmacy, Fourth Military Medical University, Xi'an, China; ^4^Department of Pharmacy, 940 Hospital of PLA Joint Logistics Support Forces, Lanzhou, China

**Keywords:** resveratrol, neurodegenerative diseases (NDs), network pharmacology, multitargets, apoptosis

## Abstract

Resveratrol is a natural polyphenol in lots of foods and traditional Chinese medicines, which has shown promising treatment for neurodegenerative diseases (NDs). However, the molecular mechanisms of its action have not been systematically studied yet. In order to elucidate the network pharmacological prospective effects of resveratrol on NDs, we assessed of pharmacokinetics (PK) properties of resveratrol, studied target prediction and network analysis, and discussed interacting pathways using a network pharmacology method. Main PK properties of resveratrol were acquired. A total of 13,612 genes related to NDs, and 138 overlapping genes were determined through matching the 175 potential targets of resveratrol with disease-associated genes. Gene Ontology (GO) function analysis and Kyoto Encyclopedia of Genes and Genomes (KEGG) pathway enrichment were performed to obtain more in-depth understanding of resveratrol on NDs. Accordingly, nodes with high degrees were obtained according using a PPI network, and AKT1, TP53, IL6, CASP3, VEGFA, TNF, MYC, MAPK3, MAPK8, and ALB were identified as hub target genes, which showed better affinity with resveratrol in silico studies. In addition, our experimental results demonstrated that resveratrol markedly enhanced the decreased levels of Bcl-2 and significantly reduced the increased expression of Bax and Caspase-3 in hippocampal neurons induced by glutamate exposure. Western blot results confirmed that resveratrol inhibited glutamate-induced apoptosis of hippocampal neurons partly by regulating the PI3K/AKT/mTOR pathway. In conclusion, we found that resveratrol could target multiple pathways forming a systematic network with pharmacological effects.

## Introduction

**Figure 1:**
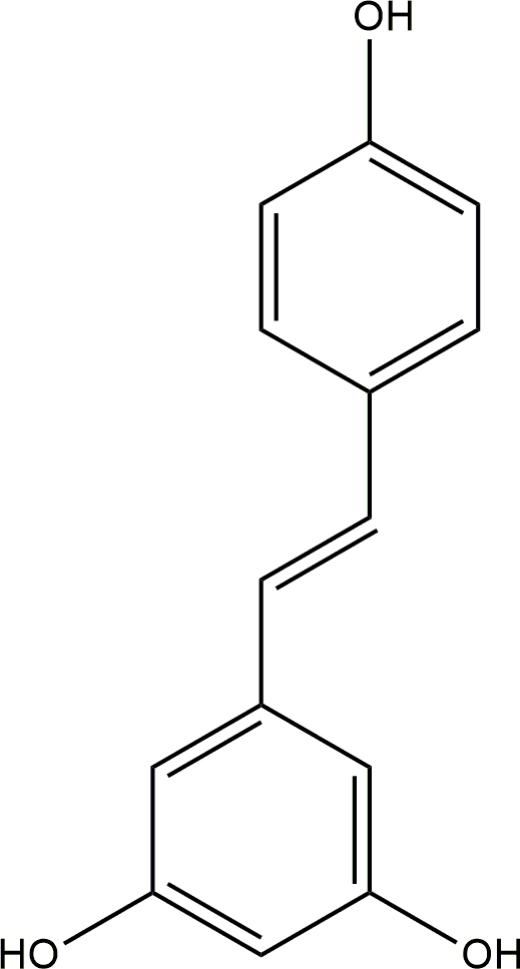
Chemical structure of resveratrol (PubChem CID: 445154).

Neurodegenerative diseases (NDs) are multifactorial debilitating disorders that are characterized by progressive dysfunction and neuronal injury, which preferentially affect the normal functioning of the brain including learning and memory (Jakaria et al., 2019). Examples of NDs are Alzheimer's diseases (AD), Parkinson's disease (PD), Huntington's diseases (HD), amyotrophic lateral sclerosis (ALS), and spinocerebellar ataxia (SCA) (Rahman et al., 2019). Notwithstanding, although the different clinical and neuropathological characteristics of each ND (Raza et al., 2018), there are several common pathological mechanisms of NDs, which are characterized by multiple targets. Most importantly, NDs are typically characterized by loss of neurons (Velmurugan et al., 2018). Some studies suggested that the pathogenesis of these kinds of diseases is the result of multiple pathological mechanisms or processes, such as glutamate excitotoxicity, oxidative stress, and abnormal apoptosis (Huang et al., 2018). Some symptomatic treatments are offered, but particular therapies have not been found owing to conventional philosophy of “one gene, one drug, one disease” (Esteves, 2017). Accordingly, the multitargeted natural products with substantial pharmacological activities are most likely to have potential advantages. Resveratrol is a natural polyphenol with a stilbene scaffold (**Figure 1**) in numerous foods including grapes, mulberries, and blueberries, which has shown many beneficial properties including antioxidant, antiinflammation, neuroprotective, and even antiaging activities. And resveratrol has revealed appealing outcomes as treatment for several NDs in preclinical studies (Penalver et al., 2018). But the mechanism by which resveratrol displays its protective function is not extremely well comprehended yet (Sanchez-Melgar et al., 2019).

Network pharmacology as an emerging discipline located on the general concepts of systems biology (Zhou et al., 2019), which was utilized to systematically evaluate pharmacological effects of multiingredient medicine (Zhang R. et al., 2019). In addition, it similarly has been provided lately for revealing the molecular mechanisms of various complicated chronic diseases, such as NDs and cardiocerebral vascular diseases (Wang W. et al., 2019). Therefore, network analysis based on lots of existing databases enables us to produce a preliminary understanding of the mechanisms by which multitarget drugs treat complex diseases.

**Figure 2:**
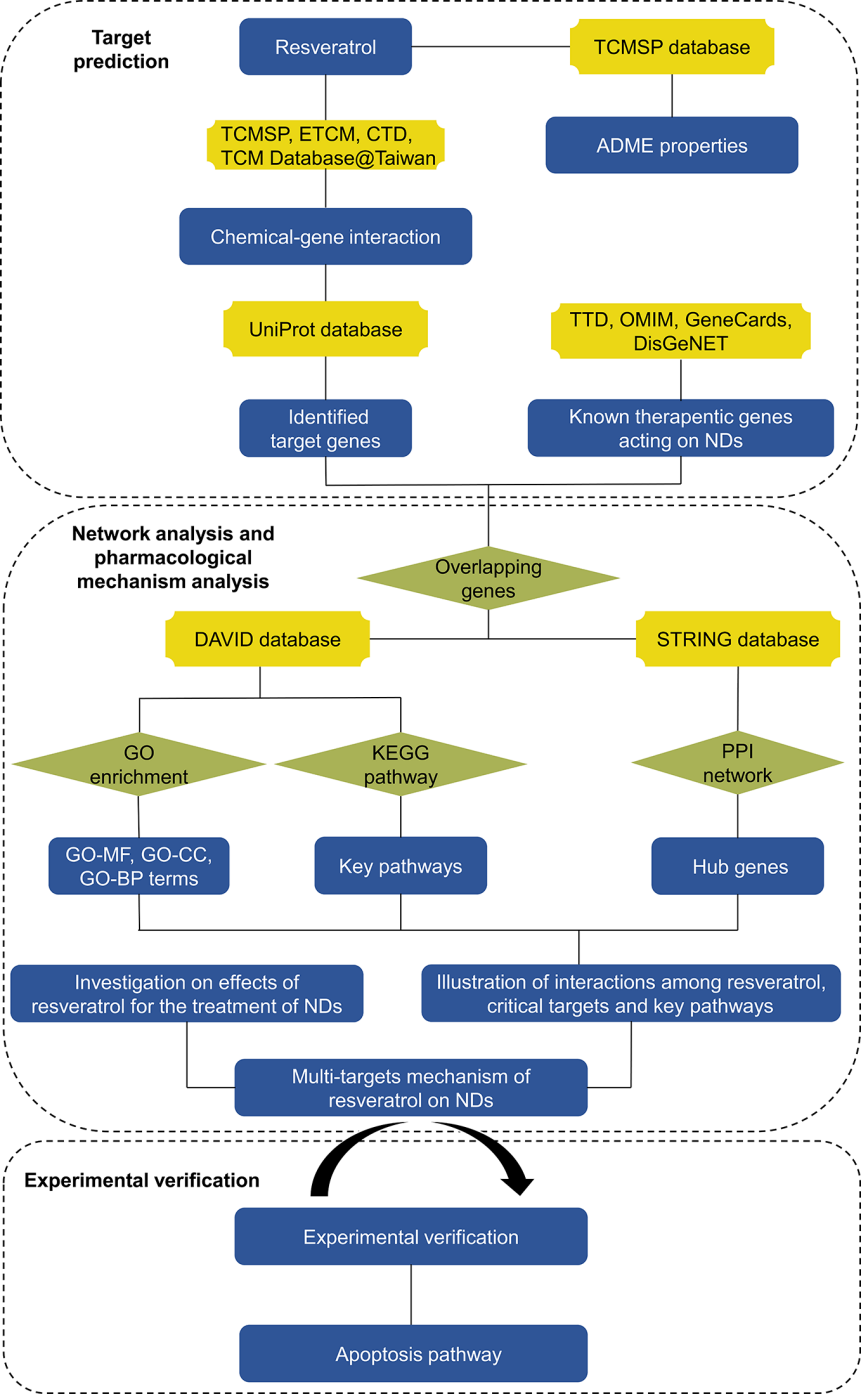
The flowchart of pharmacology analysis.

In present study, we illuminated the pharmacological actions of resveratrol on NDs systematically utilizing network pharmacology methods. Resveratrol was hypothesized to have therapeutic effects on NDs through multiple-targets mechanism. First, pharmacokinetic (PK) parameters of resveratrol were acquired from Traditional Chinese Medicine Systems Pharmacology Database (TCMSP) server. Potential candidate targets of resveratrol and therapeutic target genes of NDs were gathered respectively. Then, the potential candidate target genes were predicted *via* network pharmacology databases. In addition, multitarget of resveratrol network was constructed to supply a methodical overview. Furthermore, pivotal target genes, Gene Ontology (GO) function analysis and KEGG pathway enrichment were evaluated by STRING database and DAVID database. Finally, key targets and signaling pathways were identified by western blot. Network pharmacology analysis workflow was shown in **Figure 2**.

## Material and Methods

### Assessment of PK Parameters

PK parameters of resveratrol were acquired from TCMSP database version 2.3 (http://tcmspw.com/tcmsp.php) (Ru et al., 2014), which is a phytochemical database for TCMs or related ingredients. Meanwhile, the information of absorption, distribution, metabolism, and excretion (ADME) properties of a drug with potential biological activities also can be acquired, such as oral bioavailability (OB), drug likeness (DL), Caco-2 permeability (Caco-2), blood-brain barrier (BBB). In this study, with the chemical name “resveratrol” as the keyword, and PK properties of resveratrol were searched in the search box.

### Construction and Identification of Target Genes

All of genes associated to resveratrol were gathered from the databases: TCMSP database version 2.3 (http://tcmspw.com/tcmsp.php), TCM Database@Taiwan (http://tcm.cmu.edu.tw/) (Chen, 2011), the Comparative Toxicogenomics Database (CTD, http://ctdbase.org/) (Davis et al., 2019), and the Encyclopedia of Traditional Chinese Medicine (ETCM, www.nrc.ac.cn:9090/ETCM/) (Xu et al., 2019). Subsequently, the official names of gene were drawn from UniProt database (http://www.uniprot.org/) (Uniprot, 2019) by restricting the types to “Homo sapiens.” Then, different genes' ID terms were converted into UniProt IDs. And a resveratrol-targets relationship dataset was constructed.

### Gene Dataset Acquisition of NDs

With “NDs,” “ADs,” “PD,” “HDs,” “ALS,” and “SCA” as the keywords, then therapeutic target genes of NDs were acquired from the Therapeutic Target Database (TTD, https://db.idrblab.org/ttd/) (Wang et al., 2020), the Online Mendelian Inheritance in Man (OMIM, http://www.omim.org/) (Amberger et al., 2015), GeneCards (https://www.genecards.org/) (Fishilevich et al., 2016) and a database of gene-disease associations (DisGeNET, http://www.disgenet.org/) (Pinero et al., 2017), and just “Homo sapiens” proteins linked to NDs were selected.

### GO Function Enrichment and KEGG Pathway Analysis

A pharmacology network is comprised of nodes and edges. The entities that make up the nodes of the networks are resveratrol NDs and related target genes. The Cytoscape version 3.7.2 was used to constructed networks, which is a java based open source software (Demchak et al., 2014). Functional pathways of resveratrol related to NDs were analyzed using GO enrichment and KEGG pathways analysis based upon the database for Annotation, Visualization and Integrated Discovery (DAVID) version 6.8 (https://david.ncifcrf.gov/) (Ke et al., 2019). *P* < 0.05 suggested the enrichment degree had statistically significant and the pathway results might be essential functional mechanisms of resveratrol in the treatment of NDs.

### Construction of Target Protein-Protein Interaction (PPI) Data

The potential interprotein interactions were obtained from STRING database version 11.0 (https://string-db.org/), which is a database of known and predicted protein-protein interactions (Ge et al., 2018). The software produces scores information for each pair of protein. The higher the score, the more confident the target protein's interactions were. Thus, the potential targets of resveratrol on NDs were imported into STRING tool to acquire potential interprotein interactions. We selected a high confidence score > 0.7 with the species restricted to “Homo sapiens” (Szklarczyk et al., 2019). Then, target genes with high degree, betweenness, and closeness were selected as the hub genes of NDs.

### *In Silico* Docking Study of Resveratrol With Key Targets

A study of in silico docking of resveratrol with key targets was conducted by Autodock Vina (Trott and Olson, 2010). For this purpose, resveratrol was prepared and optimized using the PubChem database (https://www.ncbi.nlm.nih.gov/), then converted to the PDB file format. Receptor structures were downloaded from the RCSB Web site (http://www.rcsb.org/pdb) in PDB format. Before docking, the original crystal ligands and water molecules were removed from the protein-ligand complexes. Hydrogen atoms and charge were added, and default settings were selected for other parameters. The theoretical binding affinities of resveratrol to proteins are predicted based on docking score.

### Cell Culture and Treatments

The study was reviewed and approved by Ethics Committee of Animal Experimentation of the Fourth Military Medical University (Xi'an China). Primary hippocampal neurons were prepared from embryonic d15 mouse embryos. Embryonic brain tissue was mechanically triturated and centrifuged. Neurons were cultured in the atmosphere of 5%/95% CO2/air at 37°C using the Dulbecco's modified Eagle's medium (DMEM) which contains 10% fetal bovine serum, 100 U/ml of penicillin, and 100 μg/ml streptomycin. The coincubation model incorporating samples and glutamate (25 mM) was used to evaluate the protective effects of resveratrol (50 mg/ml) on glutamate-induced apoptotic cells. The equivalent volume of PBS and resveratrol were used in the control groups, respectively. To investigate the involvement of PI3K/AKT/mTOR in the effects of resveratrol, phosphatidylinositol-3-kinase (PI3K) inhibitors, LY294002 (10 μM) was added to glutamate-stimulated hippocampal neurons. All operations were repeated over three times.

### Western Blot Analysis

Primary hippocampal neurons were lysed at indicated time points with 40-μl RIPA ice-cold lysis buffer (Rockford, IL, USA) supplemented with protease inhibitor (Vazyme Biotech, Nanjing, China) for 30 min and centrifuged at 9,000 g for 10 min at 4°C. The protein concentrations were analyzed by the BCA (Rockford, IL, USA). Equal amounts of proteins were separated and transferred to a PVDF membrane (Millipore, USA). The membranes were blocked with 3% BSA in TBS/T and stained with primary antibodies (1:1,000) and antibodies for β-actin (1:10,000) overnight at 4°C. Membranes were then probed with peroxidase conjugated secondary antibody at a 1:10,000 dilution. The antigen-antibody complexes were detected with an ECL reagent (Rockford, IL, USA). Primary antibodies used for western blotting were mouse antibodies. Secondary antibodies used were peroxidase conjugated goat antimouse antibodies.

### Statistical Analysis

The data of the experiment was expressed as means ± SEM and analyzed using one-way ANOVA. *P* < 0.05 was a significant difference and *P* < 0.01 is a very significant difference.

## Results

### PK Parameters

The information of resveratrol on 12 main characteristics like Caco-2 and BBB for drug screening and evaluation (**Table 1**). Significantly, OB is the primary feature of oral medications because it plays a critical part in assessing the effectiveness of drug distribution for systemic circulation. In spite of the low bioavailability of resveratrol (its OB was calculated to be 19.07%), there are lots of evidence that resveratrol has the therapeutic potential on neurodegeneration (Yang et al., 2017). Furthermore, resveratrol has a relatively small molecular weight and moderate blood-brain barrier permeability with a BBB value of −0.01.

**Table 1:** Pharmacological and molecular properties of resveratrol.

Molecular Formula	MW	AlogP	Hdon	Hacc	OB (%)	Caco-2	BBB	DL	FASA-	TPSA	RBN	HL
C_14_H_12_O_3_	228.26	3.01	3	3	19.07	0.80	−0.01	0.11	0.49	60.69	2	–

MW, molecular weight; AlogP value represents the partition coefficient between octanol and water; Hdon and Hacc are measures of the hydrogen-bonding ability of a molecule expressed in terms of number of possible hydrogen-bond donors and acceptors, respectively; OB, oral bioavailability; Caco-2, Caco-2 permeability; BBB, blood-brain barrier; DL, drug-likeness; FASA-, fractional water accessible surface area of all atoms with negative partial charge; TPSA is a physico chemical property describing the polarity of molecules; RBN is the number of bonds which allow free rotation around themselves; HL, drug half-life.

### Potential Target Genes and Network Analysis

**Figure 3:**
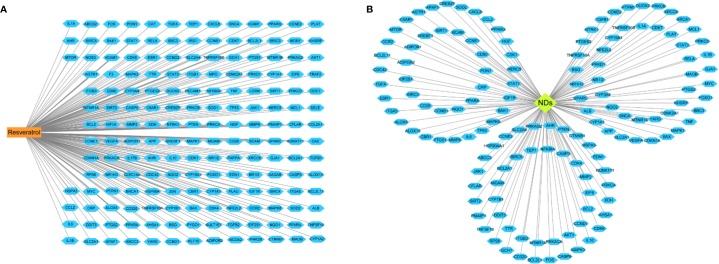
Linkage of drug, disease, and target genes. **(A)** The network of resveratrol- candidate targets. **(B)** Network of 138 common potential protein targets related to neurodegenerative diseases (NDs). The orange rectangle represents the resveratrol, green diamond means NDs and blue hexagon represent the target genes on which the drug acts.

A total of 182 candidate target genes were identified (**Supplementary Table 1**). Afterwards, seven were duplicated and therefore removed, 175 unique target genes for resveratrol remained (**Figure 3A**). Based on gene databases, we determined 13,612 genes concerned NDs (**Supplementary Table 2**). Then, 138 overlapping genes were identified through matching the potential targets of resveratrol with disease-associated genes (**Figure 3B**). Despite the different clinical and neuropathological characteristics of each ND, which are characterized by multiple targets, the underlying causes at the molecular level are almost similar (Suresh et al., 2020). Therefore, resveratrol reveled multitarget effects, which can regulate the variety of changes associated with NDs, unlike specific target drugs.

### GO Term Enrichment and Pathway Analysis

**Figure 4:**
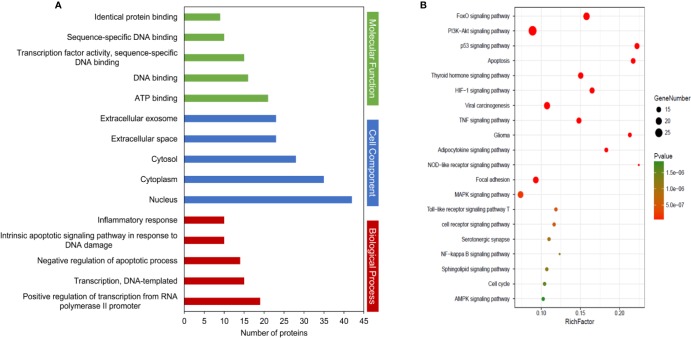
Kyoto Encyclopedia of Genes and Genomes (KEGG) pathways and Gene Ontology (GO) analyses by database for Annotation, Visualization and Integrated Discovery (DAVID) database. **(A)** GO enrichment analysis of target proteins. The number of GO entries in the functional categories of cell composition (CC), molecular function (MF), and biological process (BP) (*P* < 0.05). **(B)** KEGG pathways of target genes (*P* < 0.05).

To elucidate the function and pharmacological mechanism of resveratrol, we conducted GO enrichment and KEGG pathway analysis of the 138 determined targets. GO analysis (**Supplementary Table 3**) is determined by the biological process (BP), cell component (CC), and molecular function (MF) terms. An introduction of the GO enrichment was discovered with the leading five enriched entries in the BP, CC, and MF terms (**Figure 4A**, *P* < 0.05). Especially, the enriched BP ontologies were dominated by positive regulation of transcription from RNA polymerase II promoter, DNA-templated transcription, and negative regulation of apoptotic process, etc. The enriched MF ontologies were dominated by ATP binding, DNA binding, and so on. The nucleus accounted for the largest proportion in CC analysis (42 target genes). Then, a total of 119 significant pathways were acquired (*P* < 0.05). Among them, 20 significant pathways were showed in **Figure 4B**. As shown, FoxO, PI3K-Akt, p53 and apoptosis signaling pathways may be the interaction pathways to apply their combined results versus NDs.

### PPI Network of Target Genes

**Figure 5:**
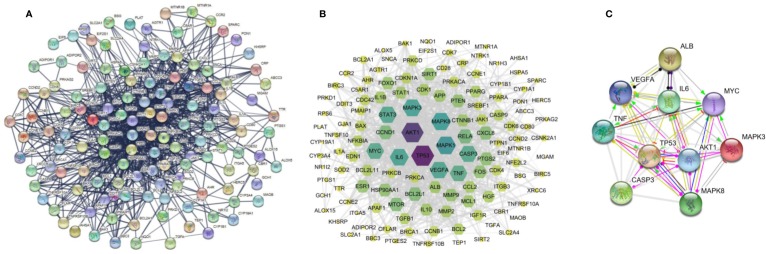
Protein-protein interaction (PPI) networks of resveratrol for the treatment of neurodegenerative diseases (NDs). **(A)** All nodes represent the relevant to genes, the edge means line thickness indicates the strength of data support. **(B)** The target genes with high degree, betweenness, and closeness. And **(C)** the predicted mode of action of 10 hub genes, “→” represents activation, “—|” means inhibition, and “—•” represents unspecified.

**Figure 6:**
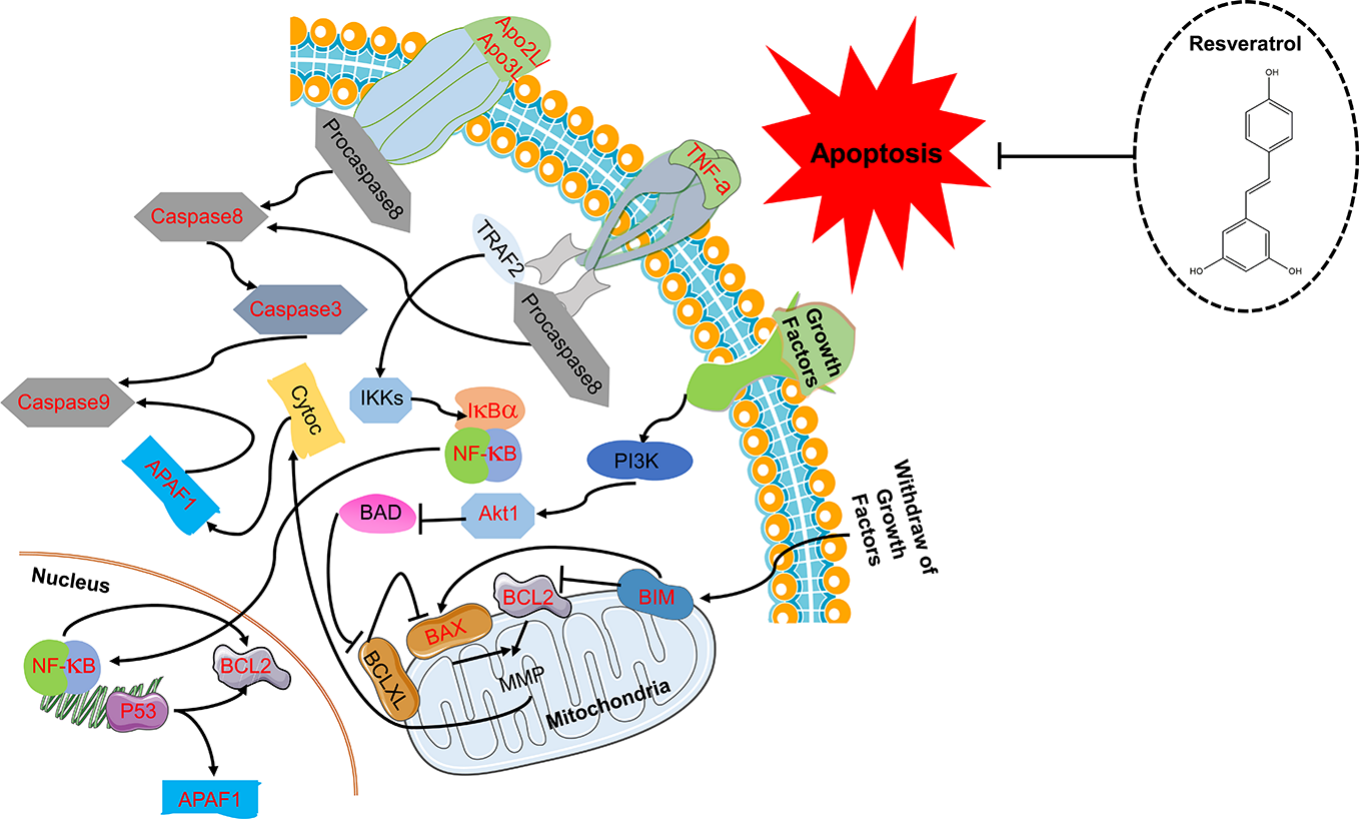
Resveratrol plays a protective role in neurodegenerative diseases (NDs) by regulating the apoptotic pathways involved in AKT1, TP53, CASP3, and TNF proteins.

The 138 target genes were submitted to STRING tool to acquire PPI relationships. We selected high-confidence target protein based on interaction with a score of > 0.7 to ensure the dependability of the study, and got the network of PPI relationships (**Figure 5A**). The targets with high degree, betweenness, and closeness were chosen as the hub genes for NDs (**Figure 5B**). And the predicted mode of action of 10 hub genes was shown in **Figure 5C**. The hub genes were including RAC-alpha serine/threonine-protein kinase (AKT1), cellular tumor antigen p53 (TP53), interleukin 6 (IL6), caspase-3 (CASP3), vascular endothelial growth factor A (VEGFA), tumor necrosis factor (TNF), myc proto-oncogene protein (MYC), mitogen-activated protein kinase 3 (MAPK3), mitogen-activated protein kinase 8 (MAPK8), and serum albumin (ALB). Among the above 10 hub proteins, such as AKT1, TP53, CASP3, and TNF, are involved in external or internal apoptotic pathways. Therefore, Resveratrol may play a protective role in NDs by regulating the apoptotic pathways involved in AKT1, TP53, CASP3, and TNF proteins (**Figure 6**).

### Molecular Docking Analysis

**Figure 7:**
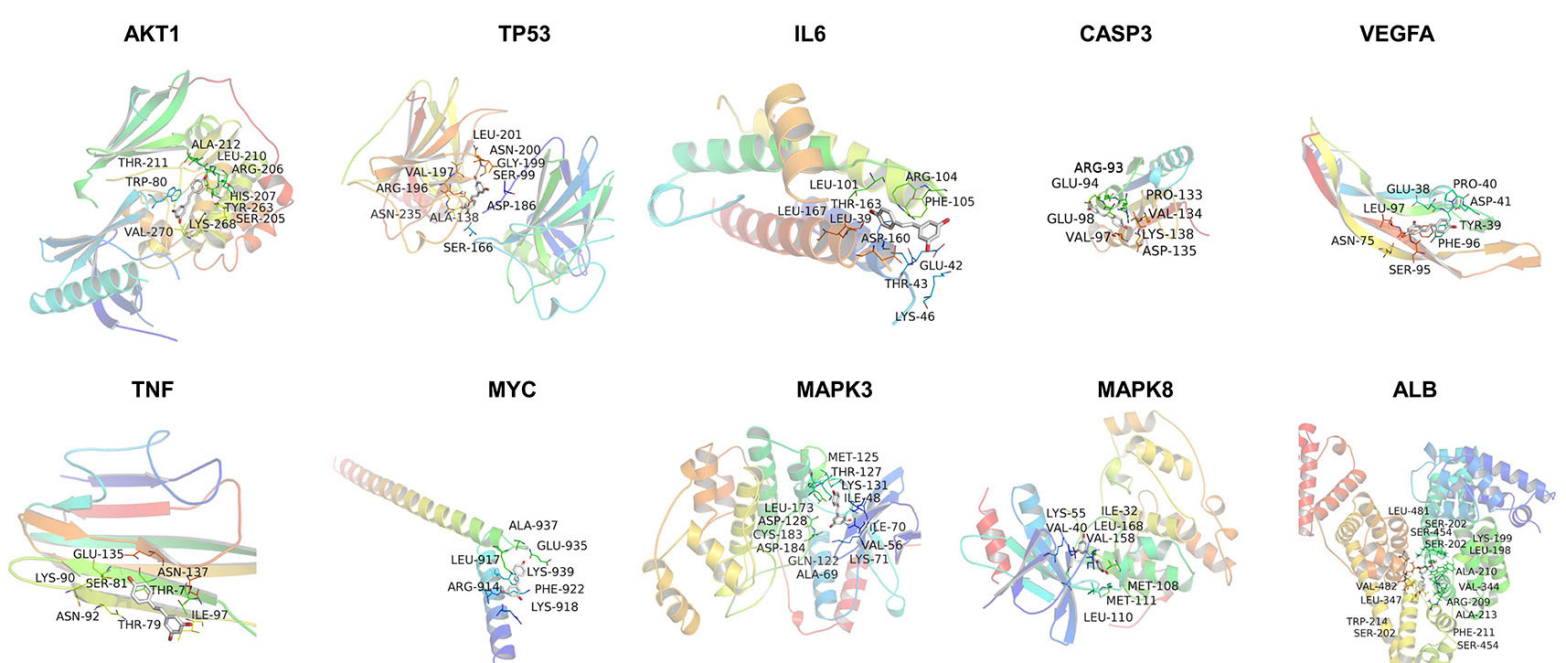
Structural interactions of resveratrol key target receptors in silico studies.

In silico studies were conducted to study the binding affinity of resveratrol with key target receptors. The results revealed that docking scores of resveratrol with AKT1, TP53, IL6, CASP3, VEGFA, TNF, MYC, MAPK3, MAPK8, and ALB ranged from −4.8 to −8.9. Particularly, resveratrol had the highest docking score with AKT1 (docking score: −8.9), which indicated that resveratrol was well located inside the binding site with AKT1. Other hub proteins also showed better affinity with resveratrol, as shown in **Figure 7** and **Table 2**.

**Table 2:** The docking scores and binding sites of resveratrol with key proteins.

Target (PDB ID)	Drug	Binding sites with the amino acid	Docking score
AKT1 (4EKL)		ALA212, THR211, TRP80, VAL270, LYS268, SER205, TYR263 HIS207, ARG206, LEU210	−8.9
TP53 (6SHZ)		VAL197, LEU201, ASN235, ALA138, SER166, ASP186, SER99, GLY199, ASN200, ARG196	−6.4
IL6 (4ZS7)		LEU101, ARG104, THR163, PHE105, LEU167, ASP160, GLU42, LEU39, THR43, LYS46	−7.1
CASP3 (6BFK)		LYS138, PRO133, ASP135, VAL134, GLU94, ARG93, GLU98, VAL97	−5.9
VEGFA (4KZN)	Resveratrol	GLU38, PRO40, LEU97, ASN75, SER95, PHE96, TYR39, ASP41	−5.2
TNF (6OOY)		GLU135, ASN137, LYS90, SER81, THR77, ASN92, THR79, ILE97	−4.8
MYC (6G6J)		ALA937, GLU935, LEU917, LYS939, ARG914, PHE922,LYS918	−5.9
MAPK3 (2ZOQ)		MET125, THR127, LYS131, ILE48, GLN122, ASP184, CYS183, ASP128, LEU173 ILE70, VAL56, LYS71, ALA69	−7.4
MAPK8 (4YR8)		LYS55, ILE32, VAL40, LEU168, VAL158, MET108, MET111, LEU110	−6.8
ALB (6QIO)		LEU481, SER202, SER454, LYS199, LEU18, ALA210, VAL344, ARG209, ALA213, PHE211, SER454, VAL482, LEU347, TRP214	−8.9

### Experimental Validation

**Figure 8:**
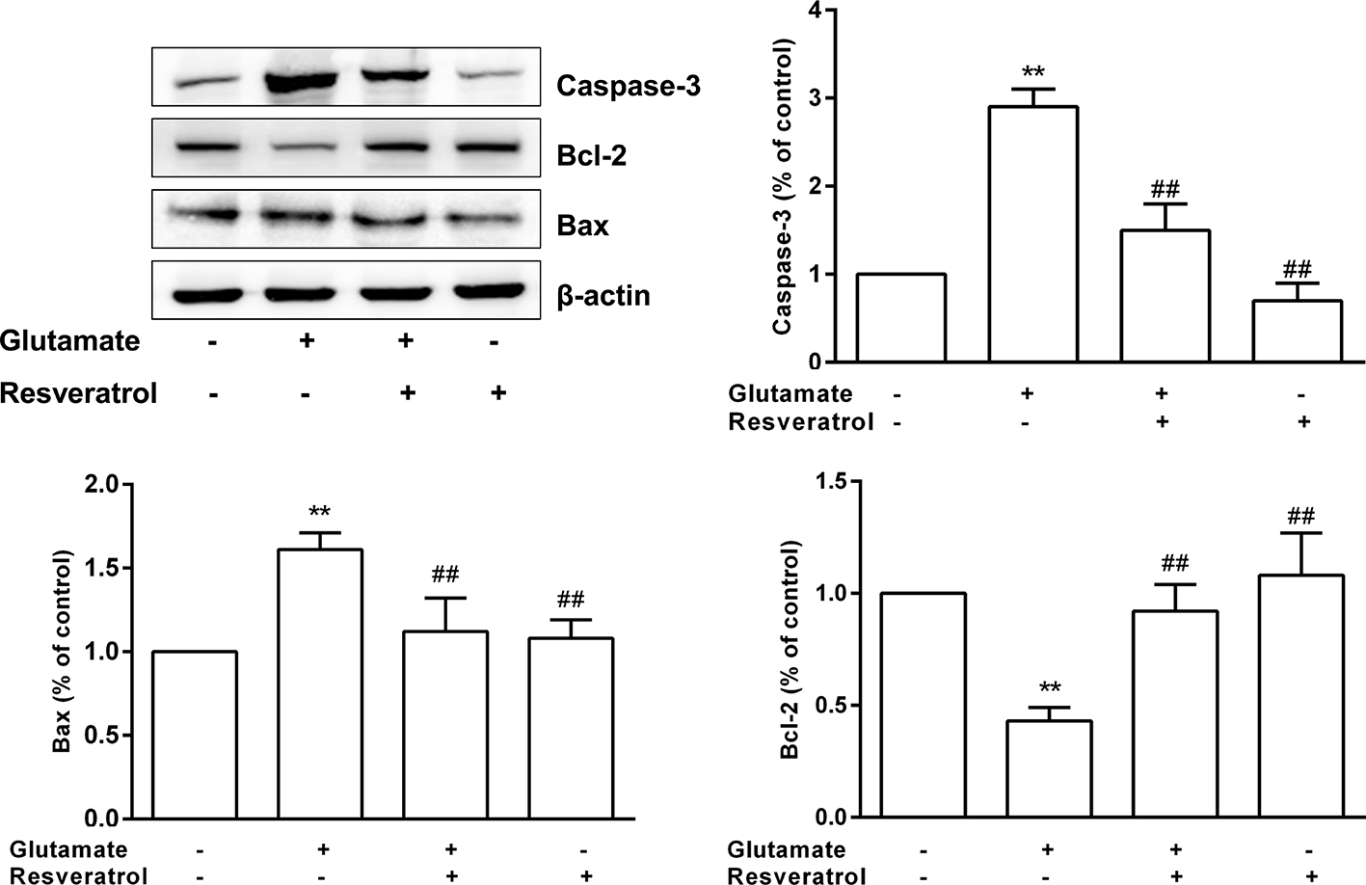
Effects of resveratrol on the expression of Caspase-3, Bcl-2, and Bax induced by glutamate exposure in hippocampal neurons. ^**^P < 0.01 compared with control; ^##^*P* < 0.01 compared with glutamate alone.

The protective effects of resveratrol on glutamate−induced injury in the culture model *in vitro* were determined. In fact, FoxO, PI3K-Akt, and p53 signaling pathways are also involved in regulating apoptosis (Akhter et al., 2014). Thus, based on the hub targets and KEGG pathway enrichment, we chose the apoptosis-related protein Bax, Bcl-2, and Caspase-3 for validation the mechanisms underlying the neuroprotective effects of resveratrol. Resveratrol markedly reinforced the decreased levels of Bcl-2 in glutamate treated hippocampal neurons (*P* < 0.01). Meanwhile, resveratrol significantly reduced the increased expression of Bax and Caspase-3 induced by glutamate exposure (*P* < 0.01, **Figure 8**).

**Figure 9:**
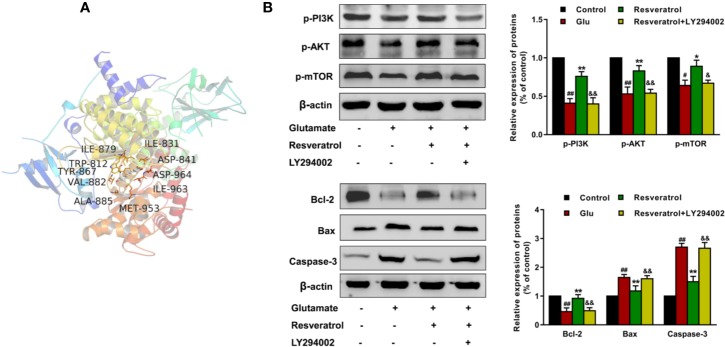
Resveratrol inhibited glutamate-induced apoptosis of hippocampal neurons by regulating the PI3K/AKT/mTOR pathway. **(A)** Structural interactions of resveratrol with PI3K (PDB ID: 6AUD) in silico studies. **(B)** Effects of resveratrol on protein expression of p-PI3K, p-AKT, p-mTOR, Bcl-2, Bax, and Caspase-3 after glutamate-induced apoptosis of hippocampal neurons. ^##^*P* < 0.01 compared with control; ^*^P < 0.05 and ^**^P < 0.01 compared with glutamate alone; ^&^*P* < 0.05 and ^&&^*P* < 0.01 compared with resveratrol treated.

The results of molecular docking showed that resveratrol was well located inside the binding site with AKT and PI3K (docking score: −7.5, **Figure 9A**), the upstream protein of AKT. It is well known that PI3K/AKT/mTOR signaling pathway is an important pathway mediating cell survival and differentiation, proliferation, apoptosis, and metastasis (Hou et al., 2018). Then we further confirmed whether resveratrol inhibited glutamate-induced apoptosis of hippocampal neurons by regulating the PI3K/AKT/mTOR pathway. The results revealed that resveratrol significantly reinforced the decreased the levels of p-PI3K, p-AKT, and p-mTOR after glutamate exposure (*P <*0.05, *P <*0.01). Additionally, LY294002 (PI3K inhibitor) significantly attenuated the effects of resveratrol (*P <*0.05, *P <*0.01), which can be observed from the protein expression of p-PI3K, p-AKT, p-mTOR and Bcl-2, Bax, and Caspase-3 (**Figure 9**). These results suggested that resveratrol plays a neuroprotective role partly by activating the PI3K/AKT/mTOR pathway to inhibit the apoptosis of neurons.

## Discussion and Conclusion

NDs are common ailments in the elderly, and they are rapidly rising in prevalence as members of society age (Bianchi et al., 2019). And NDs vary in pathophysiology, such as memory and cognitive impairments, and the ability to move, speak, and breathe is affected. There is an urgent need to search for more effective therapeutic strategies to curb the progress of NDs. Moreover, it is important to gain insight into the causes and mechanisms of each disease (Gitler et al., 2017). Current advances in network pharmacology have provided brand-new chances to elucidate the treatment of certain complex diseases with certain drugs (Zhang et al., 2016). In this study, 12 very important PK properties of resveratrol were acquired from the TCMSP database. A total of 13,612 genes relevant to NDs, and 138 overlapping genes were identified through matching the potential targets of resveratrol with disease-associated genes. To acquire a more in-depth understanding of resveratrol on NDs, we performed GO function analysis and KEGG pathway enrichment. Accordingly, genes with degree, betweenness and closeness of differential expression were gotten according utilizing a PPI network, and AKT1, TP53, IL6, CASP3, VEGFA, TNF, MYC, MAPK3, MAPK8, and ALB were identified as hub nodes. These hub targets showed better affinity with resveratrol in silico studies. In addition, our experimental results demonstrated that suggested that resveratrol plays a neuroprotective role partly by activating the PI3K/AKT/mTOR pathway to inhibit the apoptosis of neurons.

Toxicity and PK are the most important characteristics should be given priority in drug research (Zhang Y.F. et al., 2019). Resveratrol is a natural nonflavonoid polyphenol, but its intense metabolism and particularly low OB seem to limit its application in human therapeutics. However, resveratrol nanoformulations are being looked upon as a resolution to these PK issues (Summerlin et al., 2015). Additionally, Lipinski's rule of five can determine some main drug properties: the compounds with molecular weight from 180 to 500 Dalton are viewed as more druggable, AlogP value is less than 5, numbers of possible hydrogen-bond donors and acceptors are less than 5 and 10, respectively (Chagas et al., 2018). And resveratrol could be up to these standards, suggesting it could be considered a lead compound that can be structurally optimized. Thus, resveratrol is a prospect for drug development.

A growing number of studies have suggested that resveratrol is a multitarget treatment for NDs. In this study, AKT1, TP53, IL6, CASP3, VEGFA, TNF, MYC, MAPK3, MAPK8, and ALB were identified as hub genes in the PPI analysis. Similarly, several previous studies have addressed gene expression changes. The majority of these studies compared the transcriptional levels of genes in cells or animals. AKT1 exerts major influences on the regulation of cell proliferation, cell survival, and protein synthesis (Ahmad et al., 2014; Palmieri et al., 2017). In fact, resveratrol has been shown to be protective in cellular and animal models of neurodegeneration by enhancing AKT1 activity (Zhang et al., 2014; Wang et al., 2018). TP53 is a crucial protein in NDs (Goiran et al., 2018). Several studies consistently suggested that TP53-dependent apoptotic cells are detectable in specific locations in PD and AD (Checler et al., 2018). Neuroinflammation is intensively demonstrated to be related with various NDs (Qi et al., 2019). Especially, the findings of the meta-analysis demonstrated higher peripheral concentrations of IL-6 and TNF in patients with PD (Qin et al., 2016). CASP3 plays a crucial function in intrinsic and extrinsic pathways of programed cell death and in cell proliferation. Previous study reported that the administration of resveratrol could increase proinflammatory cytokine levels and inhibit apoptosis in the hippocampus (Shen et al., 2019). VEGFA is a proangiogenic factor, which also involves in neuroprotection, neurogenesis, synaptic plasticity, and modulation of inflammation and astrocyte proliferation (Harris et al., 2018; Wei et al., 2018). Recently, human induced pluripotent stem cells, which are produced from somatic cells by overexpressing four reprogramming factors (including MYC), were applying in the cellular therapy of NDs (Berry et al., 2018). ALB is the most plentiful protein in blood plasma and cerebrospinal fluid, where it contributes substantially to regulate the osmotic pressure and metabolic processes (Ts et al., 2017). Studies have demonstrated that resveratrol stabilizes the protein structure and regulates the development of fibrils along the preliminary stage of the ALB aggregation pathway (Stirpe et al., 2016). Simultaneously, MAPK is a crucial target for chronic inﬂammatory diseases such as AD (Criscuolo et al., 2017). It was reported that resveratrol reduced the upregulated protein expression of AMPK to prevent alcohol−induced neurodegeneration (Gu et al., 2018).

GO and KEGG pathway analyses were performed to better understand the interactions of the target genes. In results, GO analysis revealed that target genes were majorly related to the BPs of positive regulation of transcription from RNA polymerase II promoter, DNA-templated transcription, and negative regulation of apoptotic process, etc. The enriched MF ontologies were dominated by ATP binding, DNA binding, and so on. The nucleus accounted for the largest proportion in CC analysis. Furthermore, KEGG pathway analysis indicated that FoxO signaling pathway, PI3K-Akt signaling pathway and apoptosis, etc. may be the interaction pathways to apply their combined results versus NDs. These results followed the previous reports that above pathways participate in crucial functions in the progression of NDs (Huang et al., 2019; Qi et al., 2019; Rai et al., 2019).

NDs are typically characterized by loss of neurons. Studies have confirmed that apoptosis is an important molecular biological mechanism that is closely related to NDs (Ghavami et al., 2014). Wang N. et al. (2019) reported that resveratrol improves cognitive function of rats and reduces oxidative stress-induced neuronal damage in the frontal cortex and hippocampus by inhibiting neuronal apoptosis. In present study, among the 10 hub proteins from PPI network, such as AKT1, TP53, CASP3, and TNF, are involved in external or internal apoptotic pathways (**Figure 6**). And FoxO, PI3K-Akt, and p53 signaling pathways are also involved in regulating apoptosis. Apoptosis is a process of programmed cell death, which is vital for normal neural development (Ghavami et al., 2014). Under pathologic conditions, apoptosis also coresponsible for the loss of neurons associated with NDs (Maino et al., 2017). The proteins of Bcl-2 family, caspases and Apaf1 are main molecular components of the apoptosis program (Burek et al., 2006). Particularly, Bax gene is the first known proapoptotic member (Li et al., 2017). It has been reported that Bax promotes the release of cytochrome C by transferring it to the mitochondrial membrane, accordingly promoting downstream cell apoptosis (Kosten et al., 2008). Bcl-2 is a key member of the antiapoptotic Bcl-2 family (Garner et al., 2017). Overexpressed Bcl-2 has been demonstrated to protect nerve cells from damage by neurotoxins (Laulier and Lopez, 2012). Caspase-3 is a type of proapoptotic enzyme that plays the role of the apoptotic executor (Zhang Y. et al., 2019). Our results demonstrated that resveratrol markedly enhanced the decreased levels of Bcl-2 and significantly reduced the increased expression of Bax and Caspase-3 in hippocampal neurons induced by glutamate exposure. Then, Western blot results further confirmed that resveratrol inhibited glutamate-induced apoptosis of hippocampal neurons partly by regulating the PI3K/AKT/mTOR pathway. This further suggested that resveratrol plays a protective role against NDs through the apoptosis pathway.

In summary, resveratrol is an active and promising compound that is expected to be developed as a safe and effective multitarget drug against NDs. Our network pharmacological analysis of resveratrol predicted that the therapeutic effects of resveratrol related to NDs through mechanisms regulated by active compounds and apoptosis associated signaling pathways, such as PI3K/AKT/mTOR pathway. Further verification experiments study is necessary to explore and the key mechanisms resveratrol.

## Data Availability Statement

All datasets generated for this study are included in the article/**Supplementary Material**.

## Ethics Statement

The study was reviewed and approved by Ethics Committee of Animal Experimentation of the Fourth Military Medical University (Xi'an China).

## Author Contributions

YD, WW, and AW conceived and proposed the idea. WW, SW, YM, and TL designed the research, analyzed the data and wrote the paper. SH, LL, and YD revised the manuscript. All authors read the final manuscript.

## Funding

This work was supported by the National Natural Science Foundation of China (No. 81603385) and the Booster Plan of Xijing Hospital (XJZT18D06).

## Conflict of Interest

The authors declare that the research was conducted in the absence of any commercial or financial relationships that could be construed as a potential conflict of interest.

## Supplementary Material

Supplementary Table 1 The identified 175 potential targets of resveratrol.

Supplementary Table 2 The identified 13612 genes relevant to NDs.

Supplementary Table 3 The enriched GO terms and KEGG pathways based upon DAVID database.
